# Supplementary material for: Endothelialization of arterial vascular grafts by circulating monocytes
Source: Nat Commun. 2020 Apr 1;11:1622. doi: 10.1038/s41467-020-15361-2 (PMC7113268; doi:10.1038/s41467-020-15361-2)
Supplement: Supplementary file 1 — Supplementary Information [file 41467_2020_15361_MOESM1_ESM.pdf]

Supplementary Information

Endothelialization of arterial vascular grafts by circulating monocytes

Smith et al; *Nature Communications,* 2020.

Supplementary Figure 1

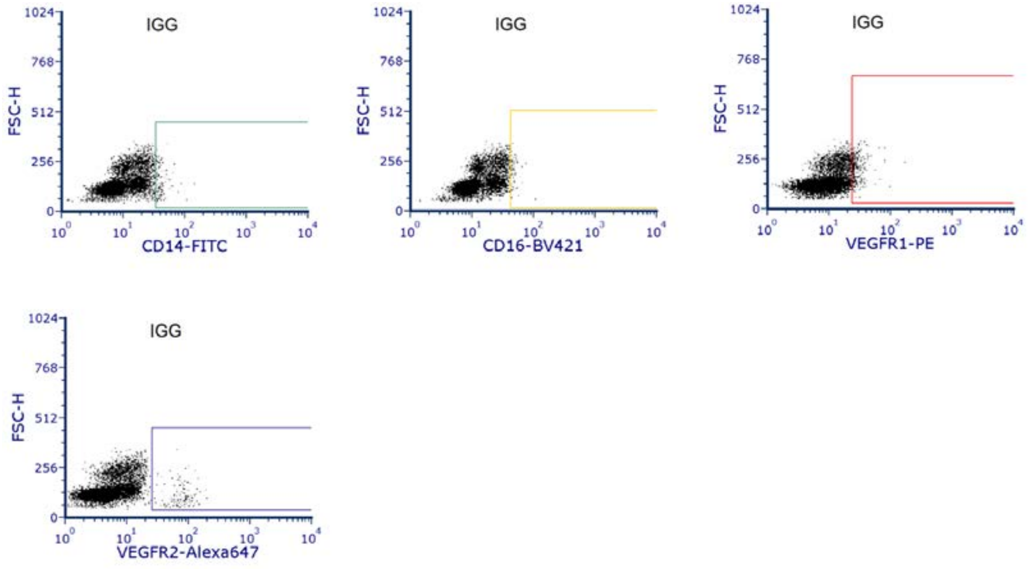

Supplementary Figure 1: Flow Cytometry Gating. For each conjugated antibody used in Figure 2, the corresponding IgG conjugated antibody was used to establish proper gating of positive/negative cell populations.

Supplementary Figure 2

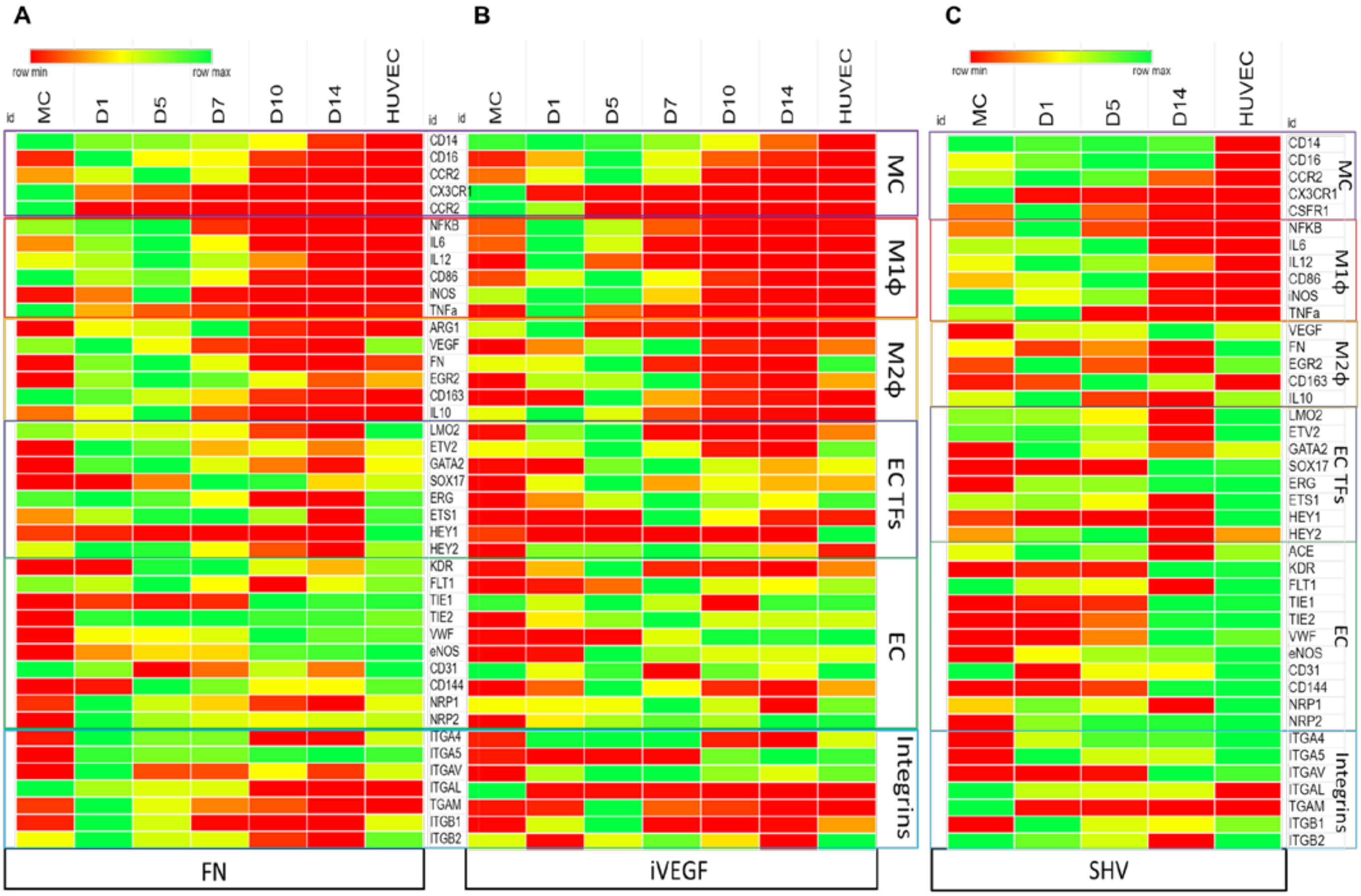

Supplementary Figure 2: Heat Maps of Gene Expression Changes during MCEC differentiation. Heat map of gene expression changes for MCEC differentiation on (A) FN, (B) iVEGF surface or when cultured on (C) SIS-Heparin-VEGF (SHV) biomaterial for monocyte genes (purple box), M1 genes (red box), M2 genes (yellow box), endothelial transcription factors (blue box), endothelial proteins (green box), and key integrins for MC and EC cells (light blue box). Color varies from red to green with red indicating the lowest and green the highest gene expression level. Each row is independently assessed. Heat map generated via Morpheus Software (https://software.broadinstitute.org/morpheus).

Supplementary Figure 3

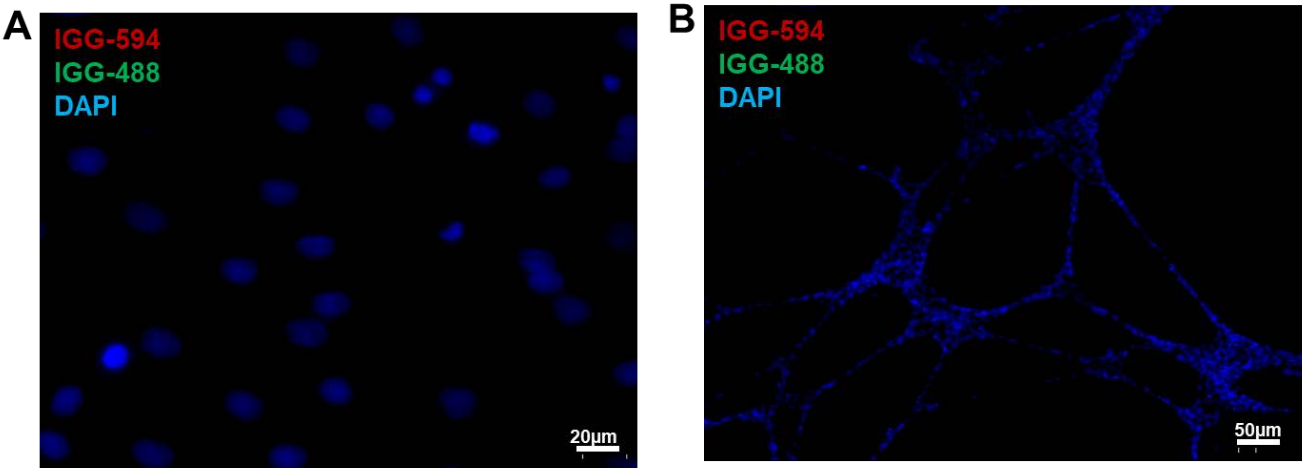

Supplementary Figure 3: Assay Controls

(A) Immunostaining of MCEC with isotype control IgG secondary antibody conjugated to Alexa 488nm or Alexa594nm. (**B**) Immunostaining of MCEC derived endothelial tubes with isotype control IgG secondary antibody conjugated to Alexa 488nm or Alexa594nm.

Supplementary Figure 4

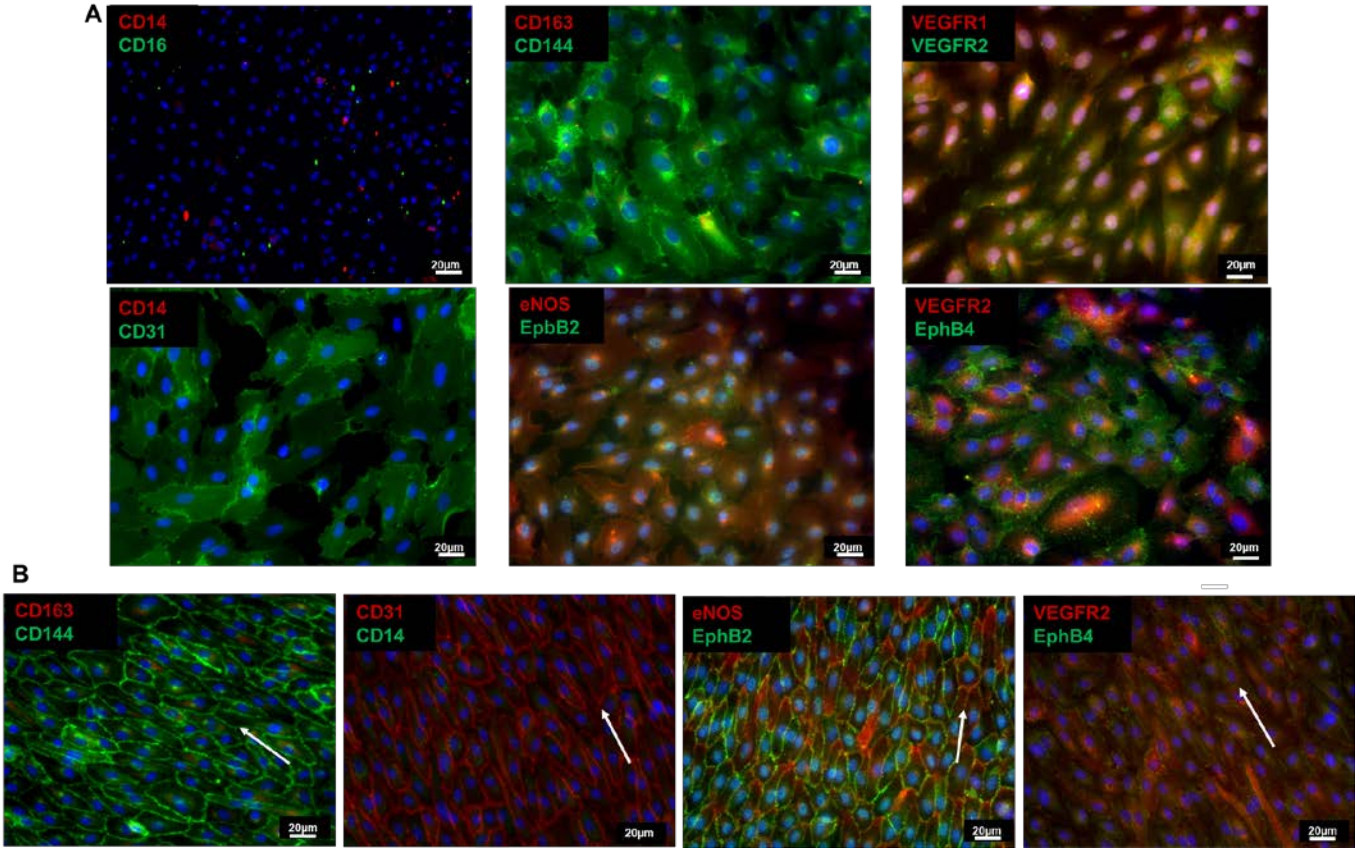

Supplementary Figure 4: Expression of MC and EC markers in HCAEC. Immunostaining of HCAEC under **(A)** static or (**B**) shear stress: VEGFR2, CD144, eNOS, EphB2, EphB4, CD31 and negative control for MC genes: CD14, CD16, CD163, VEGFR1. Scale bars as indicated.

Supplementary Figure 5

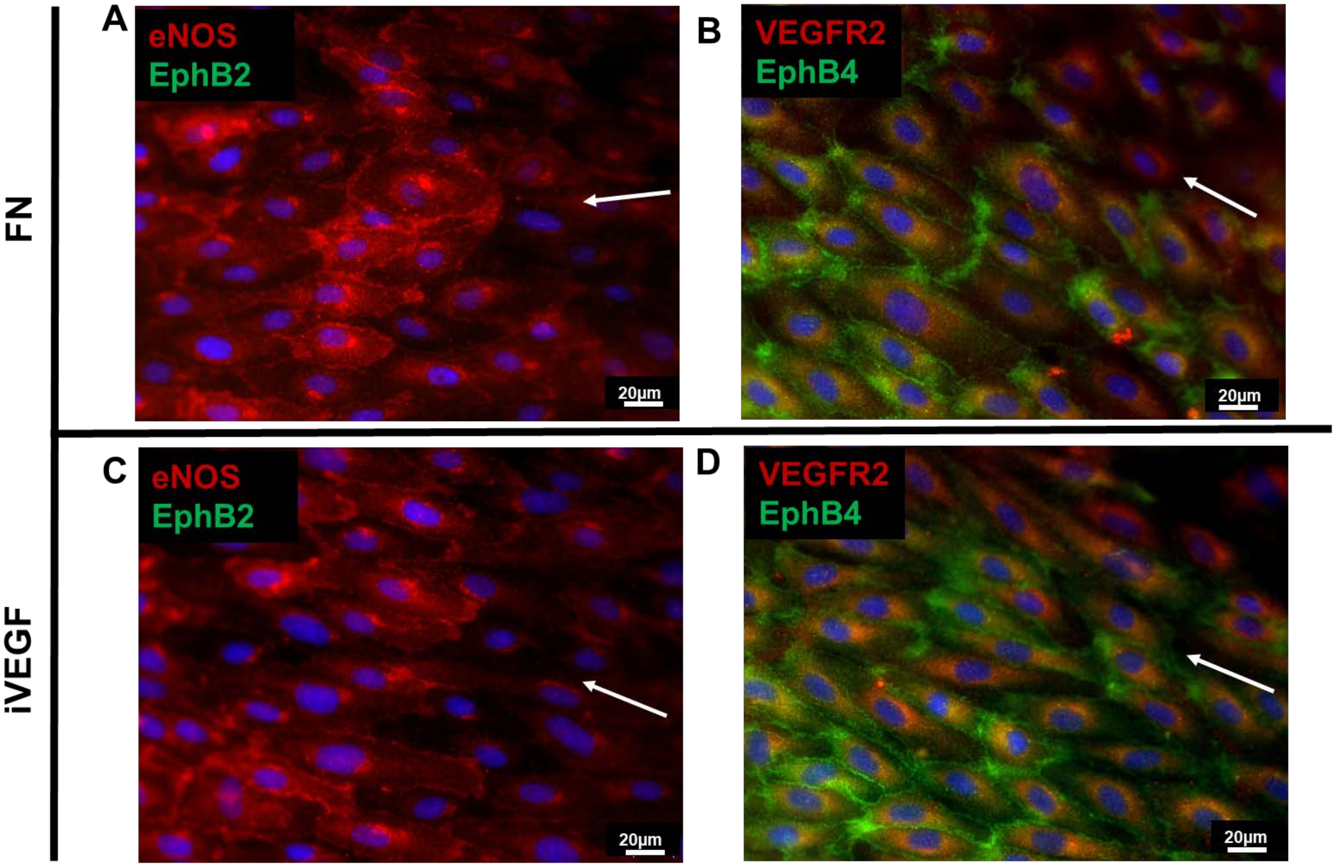

Supplementary Figure 5: MC-EC differentiation under low shear. (A, B) Differentiation on FN surface or **(C, D)** iVEGF surface under low shear (1dyn/cm^2). Immunostaining for the (A, C) arterial EC protein EphB2 (green); or (B, D) the venous EC protein EphB4 (green). White arrows indicate direction of flow. Scale bar: 20µm.

Supplementary Figure 6

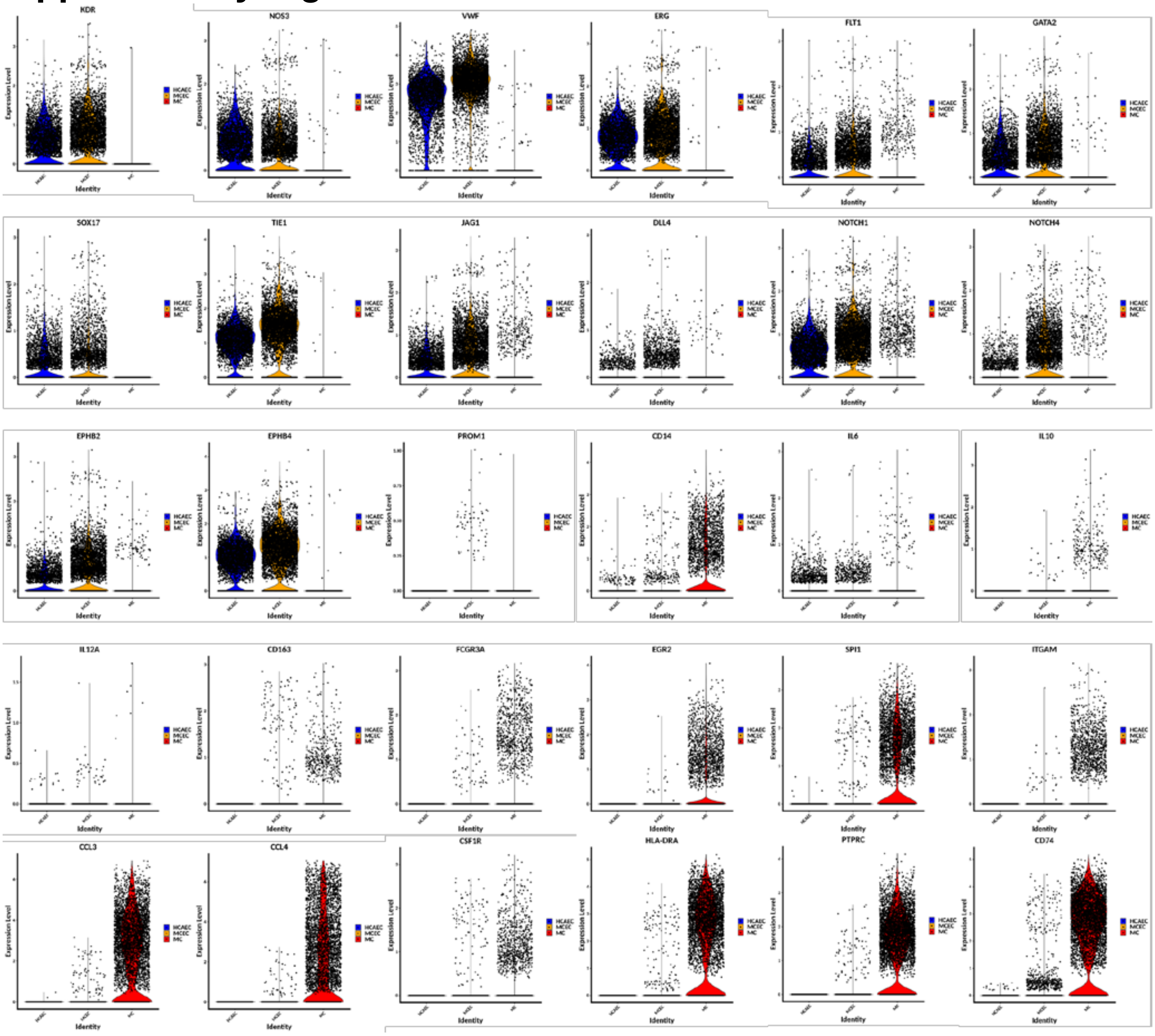

Supplementary Figure 6: Gene Expression in Single Cells from each Cell Population. Violin plots from scRNA sequencing.
