# Supplementary material for: Endothelialization of arterial vascular grafts by circulating monocytes
Source: Nat Commun. 2020 Apr 1;11:1622. doi: 10.1038/s41467-020-15361-2 (PMC7113268; doi:10.1038/s41467-020-15361-2)
Supplement: Supplementary file 3 — Reporting Summary [file 41467_2020_15361_MOESM3_ESM.pdf]

nature research

Corresponding author(s): Stelios T. Andreadis

Last updated by author(s): 03/01/2020

Reporting Summary

Nature Research wishes to improve the reproducibility of the work that we publish. This form provides structure for consistency and transparency in reporting. For further information on Nature Research policies, see Authors & Referees and the Editorial Policy Checklist.

Statistics

For all statistical analyses, confirm that the following items are present in the figure legend, table legend, main text, or Methods section.

n/a	Confirmed	
☐	☒	The exact sample size (*n*) for each experimental group/condition, given as a discrete number and unit of measurement
☐	☒	A statement on whether measurements were taken from distinct samples or whether the same sample was measured repeatedly
☐	☒	The statistical test(s) used AND whether they are one- or two-sided<br>*Only common tests should be described solely by name; describe more complex techniques in the Methods section.*
☒	☐	A description of all covariates tested
☐	☒	A description of any assumptions or corrections, such as tests of normality and adjustment for multiple comparisons
☐	☒	A full description of the statistical parameters including central tendency (e.g. means) or other basic estimates (e.g. regression coefficient) AND variation (e.g. standard deviation) or associated estimates of uncertainty (e.g. confidence intervals)
☐	☒	For null hypothesis testing, the test statistic (e.g. *F*, *t*, *r*) with confidence intervals, effect sizes, degrees of freedom and *P* value noted<br>*Give P values as exact values whenever suitable.*
☒	☐	For Bayesian analysis, information on the choice of priors and Markov chain Monte Carlo settings
☒	☐	For hierarchical and complex designs, identification of the appropriate level for tests and full reporting of outcomes
☒	☐	Estimates of effect sizes (e.g. Cohen's *d*, Pearson's *r*), indicating how they were calculated

Our web collection on statistics for biologists contains articles on many of the points above.

Software and code

Policy information about availability of computer code

Data collection	*Provide a description of all commercial, open source and custom code used to collect the data in this study, specifying the version used OR state that no software was used.*
Data analysis	Excel 2017 and Graphpad PRISM was used to perform data analysis of cell capture, spreading, acLDL uptake, NO2 production, and qPCR analysis.<br>BioRad Software CFX Manager Ver 3.1 was used for qPCR cycle determination.<br>R analysis package Seurat144.The data was normalized using Seurat's LogNormalize, with a scale factor of 10,000. Integration of all three datasets was performed using Seurat's FindIntegrationAnchors followed by IntegrateData. Principal Component Analysis (PCA), cluster analysis and Uniform Manifold Approximation and Projection (UMAP) dimensionality reduction were applied to the integrated dataset.

For manuscripts utilizing custom algorithms or software that are central to the research but not yet described in published literature, software must be made available to editors/reviewers. We strongly encourage code deposition in a community repository (e.g. GitHub). See the Nature Research guidelines for submitting code & software for further information.

Data

Policy information about availability of data

All manuscripts must include a data availability statement. This statement should provide the following information, where applicable:
- Accession codes, unique identifiers, or web links for publicly available datasets
- A list of figures that have associated raw data
- A description of any restrictions on data availability

All relevant raw data (Fig. 2b, 2c, 2e, 2f, 2g, 4c, 4e, 7a, 8a, 9) is provided as a source data excel sheet. The RNAseq data discussed in this publication have been deposited in NCBI's Gene Expression Omnibus and are accessible through GEO Series accession number GSE143353. (https://www.ncbi.nlm.nih.gov/geo/query/acc.cgi?acc=GSE143353)

Field-specific reporting

Please select the one below that is the best fit for your research. If you are not sure, read the appropriate sections before making your selection.

☒ Life sciences ☐ Behavioural & social sciences ☐ Ecological, evolutionary & environmental sciences

For a reference copy of the document with all sections, see nature.com/documents/nr-reporting-summary-flat.pdf

Life sciences study design

All studies must disclose on these points even when the disclosure is negative.

Sample size	Sample size is noted throughout the study. Sample sizes were chosen to reduce variability.
Data exclusions	No data was excluded.
Replication	All differentiation experiments were repeated multiple times for reproducibility. In addition, a second researcher performed the same set of experiments and obtained similar results (all of which are used within this study).
Randomization	Randomization was not required for this study. There is clearly defined variants with control groups as necessary.
Blinding	Blinding was used only in PBMNC collection. "Cones" were purchased without identifiers.

Reporting for specific materials, systems and methods

We require information from authors about some types of materials, experimental systems and methods used in many studies. Here, indicate whether each material, system or method listed is relevant to your study. If you are not sure if a list item applies to your research, read the appropriate section before selecting a response.

Materials & experimental systems

n/a	Involved in the study	
☐	☒	Antibodies
☐	☒	Eukaryotic cell lines
☒	☐	Palaeontology
☐	☒	Animals and other organisms
☒	☐	Human research participants
☒	☐	Clinical data

Methods

n/a	Involved in the study	
☒	☐	ChIP-seq
☐	☒	Flow cytometry
☒	☐	MRI-based neuroimaging

Antibodies

Antibodies used

CD14-FITC, (BD Biosciences), Catalog No.345784, Clone MϕP9
CD16-BV421,(BD Biosciences), Catalog No.562878, Clone 3G8
VEGFR2-Alexa Fluor-647, (BD Biosciences), Cat No.:560495, Clone: 89106
VEGFR1-PE (Miltenyi Biotech),Cat No. 130-108-928, Clone: REA569
VEGFR1 (Thermo Fisher), Cat #MA5-32045, Clone (SY09-09)
VEGFR2 (Thermo Fisher), Cat #MA5-15556, Clone 4B4
smooth muscle alpha actin (Thermo Fisher), Cat #MA1-06110, Clone 1A4
CD144 (Cell Signalling Technologies), Cat No. 2500S, Clone D87F2
CD16 (Thermo Fisher), Cat #MA1-10112, Clone 3G8
CD14 (Abgent), Cat# AP6294A, Clone (N-term)
CD163 (Santa Cruz) Clone (GHI/61), Cat: sc-20066
CD38 (1:200, Abcam), [Clone: EPR4106] Cat No.(ab108403)
EGR2 (Abcam), [Clone: OTI1F10], Cat No. (ab156765)
CD31 (Thermo Fisher) Cat #MA5-13188, Clone JC/70A
phosphorylated-eNOS (BD Biosciences) Cat No. 612393, Clone:pS1177

Validation

All Endothelial antibodies (VEGFR2-647, VEGFR1-PE, VEGFR1, VEGFR2, CD144, eNOS) were validated using endothelial cell controls (HUVEC and HCAEC) against MC (day 0) in addition to the validation performed by the companies.
MC antibodies (CD14-FITC, CD16-BV421, CD14, CD16, CD163, CD38, EGR2) were validated by staining endothelial cells (HUVEC and HCAEC) as negative controls in addition to validation done by each specific company.

Eukaryotic cell lines

Policy information about cell lines

Cell line source(s)	ATCC: NIH-3T3, RAW 264
Authentication	None of the cell lines were authenticated within our lab. We rely on previously authenticated cell lines.
Mycoplasma contamination	All cell lines were tested negative for mycoplasma contamination.
Commonly misidentified lines (See ICLAC register)	NA

Animals and other organisms

Policy information about studies involving animals; ARRIVE guidelines recommended for reporting animal research

Laboratory animals	A mixed breed (Dorset cross) of female sheep, 2–4 yrs of age, 30–50kg (New Life Pastures, Varysburg, NY)
Wild animals	This study did not include wild animals.
Field-collected samples	This study did not use field collected samples
Ethics oversight	The University at Buffalo Institutional Animal Care and Use Committee provided oversight over animal experimentation.

Note that full information on the approval of the study protocol must also be provided in the manuscript.

Flow Cytometry

Plots

Confirm that:

- [x] The axis labels state the marker and fluorochrome used (e.g. CD4-FITC).
- [x] The axis scales are clearly visible. Include numbers along axes only for bottom left plot of group (a 'group' is an analysis of identical markers).
- [x] All plots are contour plots with outliers or pseudocolor plots.
- [x] A numerical value for number of cells or percentage (with statistics) is provided.

Methodology

Sample preparation	Flow cytometry was performed on 3 different MC isolation methods, buffy coat PBMNCs, fibronectin (FN) captured PBMNCs, and iVEGF captured PBMNCs. FN surfaces were prepared using human FN (Thermo Fisher) at 10μg/mL in PBS overnight at 4˚C. Surfaces with VEGF were prepared as just discussed. For FN and iVEGF surfaces, buffy coat PBMNCs were allowed to adhere to either FN or iVEGF surfaces for 1 hr at 37˚C/5% CO2 in the absence of serum; gently washed with PBS to remove unbound cells; and then gently mechanically removed from the surface and fixed with 4% paraformaldehyde.
Instrument	BD Fortessa X-20
Software	FCS Express software suite (DeNovo Software; Naples, CA)
Cell population abundance	We did not perform cell sorting. We verified populations using single antibody staining.
Gating strategy	Gating was minimal to show all cells from the isolations. Gating for positive or negative populations was determined via use of conjugated IGG controls as specified.

- [x] Tick this box to confirm that a figure exemplifying the gating strategy is provided in the Supplementary Information.
